# The Advantageous Impact of Telestroke: Global Insights and Implications for Africa: A Scoping Review of Literature

**DOI:** 10.1155/srat/5635369

**Published:** 2025-03-20

**Authors:** Jonathan Kissi, Caleb Annobil

**Affiliations:** ^1^Department of Health Information Management, School of Allied Health Sciences, University of Cape Coast, Cape Coast, Ghana; ^2^Department of Management Science in Global Affairs, Schwarzman College, Tsinghua University, Beijing, China

**Keywords:** stroke, stroke management, telestroke, telestroke impact, telestroke in Africa, telestroke limitations

## Abstract

**Introduction:** Stroke is a leading global contributor to mortality and disability. Low- and middle-income countries are disproportionately affected and account for 87% of stroke-related disabilities and 70% of stroke-related fatalities. The challenges of stroke care accessibility in Africa are compounded by financial constraints, geographical barriers, and inadequate healthcare infrastructure, necessitating the adoption of innovative models such as telestroke. Telestroke is a critical component of modern stroke care systems. Telestroke enables real-time remote assessments, optimizes patient triage and hospital transfers, improves the efficiency of thrombolysis administration, and enhances poststroke management by mitigating logistical and mobility-related challenges. This demonstrates telestroke's potential to expand access to specialized stroke care, improve functional outcomes, and address critical gaps in stroke management within underserved regions such as Africa. This paper assesses the advantageous impact of telestroke on stroke management, with the aim of drawing global insights for Africa.

**Methodology:** This scoping review adhered to the Preferred Reporting Items for Systematic Review and Meta-Analyses (PRISMA) guidelines. A comprehensive search was conducted across ProQuest, PubMed, Google Scholar, and Scopus to identify peer-reviewed studies published in English from 2017 to 2023. This ensured the inclusion of the most recent advancements in telestroke research.

**Results:** The initial literature search retrieved 881 articles, of which 143 duplicates (16.2%) and 58 non-English studies (6.6%) were removed, followed by the exclusion of 451 nonpeer-reviewed publications (51.2%) and 128 articles (14.5%) unrelated to the study area, leaving 101 studies (11.5%) for full-text review. After further screening, 70 studies were excluded for not aligning with the study's title, objectives, or key search terms. This resulted in 31 studies (3.5%) being included in the final analysis, with 21 studies originating from outside Africa. The limited availability of high-indexed, peer-reviewed African telestroke studies highlighted a research gap, impacting the generalizability of findings.

**Conclusion:** Telestroke has demonstrated significant benefits in stroke management, including improved functional outcomes, reduced onset-to-treatment time, enhanced diagnostic accuracy, and increased healthcare accessibility, particularly in medically underserved regions. However, its implementation in Africa faces challenges related to ethical concerns, technological infrastructure, regulatory inconsistencies, financial sustainability, and limited clinician buy-in. This necessitates strategic interventions such as standardized regulatory frameworks, network expansion, sustainable financing, capacity-building, and the integration of cost-effective imaging technologies to enhance stroke care delivery across the continent.

## 1. Introduction

Cerebrovascular accident, commonly known as stroke, is a leading contributor to worldwide mortality and a substantial contributor to incapacitation. The global burden of stroke in 2019 was 143 million recorded cases [1]. Preponderantly, low- and middle-income countries bear the most substantial global burden of strokes. Swift identification and treatment of afflicted individuals, particularly in remote or rural locales, are imperative measures to mitigate ensuing complications [2]. Approximately 87% of disability arising from strokes and 70% of stroke-related fatalities occur in low-income and middle-income nations. On a global scale, an estimated one in four adults is projected to experience a stroke during their lifetime. Africa may record stroke incidence rates two to three times higher than those observed in Western Europe and the United States, coupled with elevated stroke prevalence [3]. Africa shoulders the predominant burden of hypertension, identified as the most robust and prevalent modifiable risk factor for strokes. In the African context, stroke emerges as a pivotal health determinant, historically deemed infrequent due to resource constraints hindering comprehensive community-based studies for accurate burden assessment [4].

Primary impediments to healthcare service accessibility in Africa encompass the financial outlay associated with services, the geographical distance between health facilities and the residences of service users, and prolonged waiting time at healthcare facilities [5]. In a concurrent mixed methods investigation carried out in Zambia's challenging-to-reach districts of Kaputa and Ngabwe, findings revealed that residents had to travel approximately 12 km to reach healthcare services. The study indicated that patients were inclined to remain at home rather than seek health services when lacking transportation fares. Beyond the distance to health centers, participants raised concerns about the poor condition of roads, exacerbated during rainy seasons, rendering modes of transport such as motorcycles and cars challenging [6].

Telestroke is a pivotal element within stroke systems of care, offering a means to swiftly assess individuals exhibiting acute stroke symptoms in remote or underserved regions. Telestroke serves as a crucial tool in identifying optimal candidates for hospital transfer. Its application effectively limits unwarranted transfers of patients, particularly those with mild stroke syndromes or stroke mimics, who can be appropriately managed at local facilities [7]. Telestroke is the use of remote information and communication technologies to enable the delivery of stroke care for patients, facilitating the remote practice of physicians as stroke patients concurrently receive care from a distance [8]. The use of telestroke for managing strokes is considered safe and demonstrates improvement in functional outcomes [9]. Telestroke could potentially facilitate prompt consultations by stroke neurologists to determine the appropriateness of administering thrombolysis therapy. Such an approach may enhance the utilization rates and efficiency of the thrombolysis administration timeline in the management of stroke in diabetic patients [10]. The integration of telestroke services into transitional and poststroke care has significant potential to bridge critical gaps in the continuum of care for stroke survivors, who often encounter challenges distinct from those faced by individuals with other chronic conditions. Telestroke alleviates transportation challenges, particularly for patients subject to driving restrictions, thereby enhancing accessibility and continuity of care in this vulnerable population. By addressing mobility limitations and reducing dependence on specialized equipment required to access physical clinic spaces, telestroke minimizes logistical barriers to care [11]. Telestroke has demonstrated a high level of diagnostic accuracy in differentiating stroke from stroke mimics. A retrospective analysis of all telestroke evaluations conducted within the Ochsner Health Telestroke program in the United States from April 2015 to April 2016 reported an 85% accuracy rate across 874 assessments [12]. These findings underscore the benefits of telestroke and highlight the importance of examining global insights to inform its implementation in Africa. A comprehensive assessment of international experiences with telestroke may provide valuable lessons for Africa, facilitating the adoption of best practices, addressing region-specific challenges, and enhancing the scalability and sustainability of telestroke services within the continent's healthcare systems. This paper, therefore, assesses the advantageous impact of telestroke services on stroke management, encompassing clinical outcomes, patient satisfaction, and healthcare accessibility, with the goal of drawing global insights for Africa.

## 2. Methodology

### 2.1. Search Strategy and Selection Criteria

Following the Preferred Reporting Items for Systematic Review and Meta-Analyses (PRISMA) guidelines, an extensive search for relevant data was conducted across multiple databases, including ProQuest, PubMed, Google Scholar, and Scopus (see Table 1). The search is aimed at identifying articles published from 2017 to 2023 to ensure the analysis reflects the most recent advancements and current trends in the field of telestroke. At the time the study was conducted, 2023 was the current year, and limiting the search to this timeframe allowed for the incorporation of up-to-date findings and methodologies. This approach helped to maintain the relevance of the research and address contemporary issues that could influence the study's outcomes. The search employed specific key terms such as “Stroke,” “Telestroke,” “Telestroke Impact,” “Stroke Management,” “Healthcare Accessibility,” and “Patient Satisfaction.” To ensure methodological rigor, data extraction and synthesis were carried out independently by the authors. Strict inclusion criteria were applied, focusing on publications in the English language. The review prioritized articles with complete publications, allowing for a comprehensive critical assessment of their results and discussions. The adherence to PRISMA guidelines ensures transparency and systematic reporting in this review. The inclusion and exclusion criteria were judiciously chosen to encompass studies directly aligned with the key search terms and to maintain a rigorous standard, requiring the availability of full-text articles for in-depth evaluation. This meticulous approach enhances the reliability and relevance of the synthesized data in advancing our understanding of the impact of telestroke on stroke management, healthcare accessibility, and patient satisfaction.

### 2.2. Inclusion and Exclusion Criteria

The following inclusion and exclusion criteria were applied during the study selection process:

*Inclusion criteria:* (i) Studies published in the English language. (ii) Studies published from 2017 to 2023. (iii) Peer-reviewed published studies with high indexing. (iv) Studies that address the title, key search terms, and the objective of the study.

*Exclusion criteria:* Studies published before 2017. (ii) Grey literature, dissertations, and unpublished studies. (iii) Nonpeer-reviewed studies. (iv) Studies that do not align with the title, key search terms, and the objective of the study.

The inclusion and exclusion criteria were carefully established to ensure the selection of relevant and high-quality studies for this review. Only studies published in English between 2017 and 2023 were considered, as 2023 was the current year at the time of writing. Limiting the review to this period allowed for the integration of the most recent literature on the topic. Peer-reviewed studies with high indexing that aligned with the study's title, key search terms, and objectives were included to ensure academic rigor and relevance. Conversely, studies published before 2017, grey literature, dissertations, unpublished studies, and nonpeer-reviewed research were excluded to maintain methodological robustness and focus on validated findings.

## 3. Results

**Figure 1:**
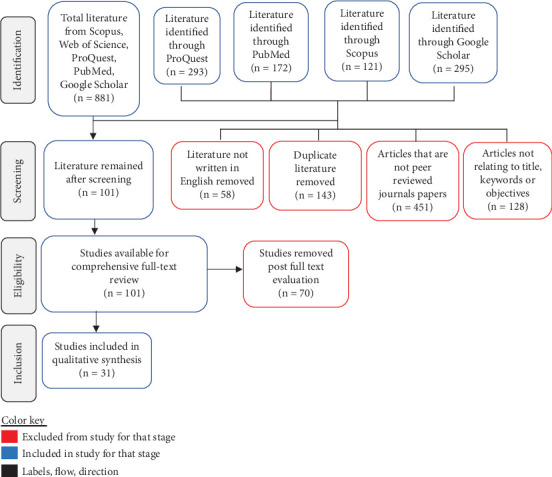
PRISMA flow for the process of article inclusion and exclusion.

The initial literature search retrieved a total of 881 articles from four electronic databases. After eliminating 143 duplicates (16.2%) and excluding 58 non-English studies (6.6%), the pool was further refined by removing 451 nonpeer-reviewed publications (51.2%) and articles not relating to the study area (128, (14.5%) removed. This process culminated in 101 studies (11.5%) available for comprehensive full-text review. Subsequent screening of the 101 studies resulted in the exclusion of 70 studies that did not align with the study's title, objectives, or key search terms. Ultimately, 31 studies, which represent 3.5% of the total retrieved articles (881), met the inclusion criteria and were incorporated into this study. This is shown in Figure 1.

**Figure 2:**
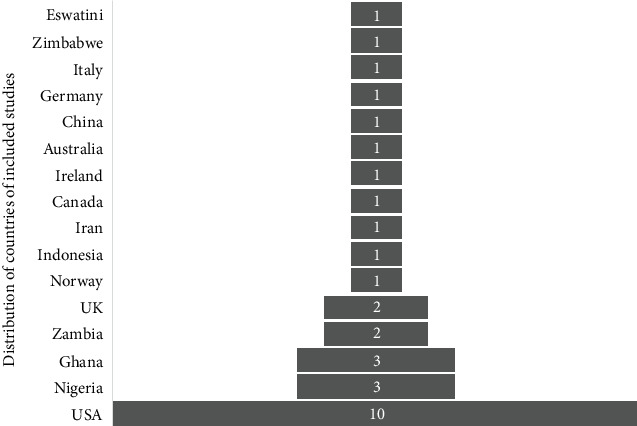
Distribution of countries based on their respective number of included articles.

**Figure 3:**
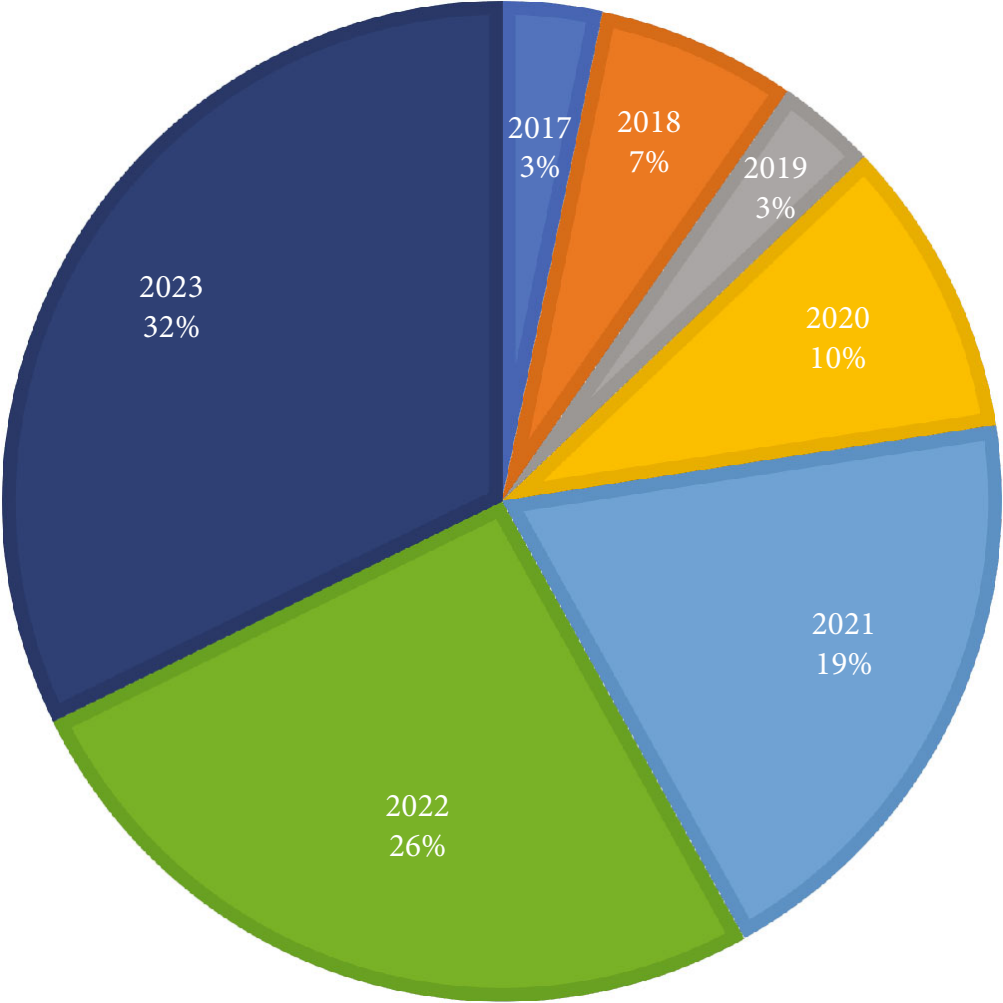
Year of publication and percentage influence of articles included in the study.

The included studies shed light on the beneficial impact of telestroke services in stroke management, clinical outcomes, patient satisfaction, and healthcare accessibility. These findings contribute to the growing body of knowledge on the positive effects of telestroke interventions in the field of stroke care.  Table 2 shows the summary characteristics of included studies for this research while Figure 2 shows the country distribution of the included articles for the study. This study included a total of 31 peer-reviewed papers, of which 21 were from countries outside Africa. The limited availability of high-indexed, peer-reviewed literature on telestroke within the African context highlighted a significant research gap. The primary objective was to examine global perspectives on the benefits of telestroke and derive lessons applicable to Africa. Studies from Africa were primarily used to contextualize the introduction and to identify potential barriers to telestroke implementation across the continent. Consequently, the limited representation of African-specific studies may have influenced the generalizability of the findings to healthcare systems within the region.  Figure 3 shows the years of publication and percentage influences of the articles included in the study. The study focused on literature published between 2017 and 2023, as 2023 was the most recent year at the time of the research. This timeframe was selected to ensure the inclusion of the most up-to-date and relevant information, enhancing the study's applicability to current advancements and trends in telestroke research.

## 4. Discussion

Stroke, a significant contributor to global mortality and disability, recorded 143 million cases in 2019 [1]. Low- and middle-income countries, particularly in Africa, bear a substantial burden, with 87% of stroke-related disabilities and 70% of fatalities occurring in these regions [2]. Africa faces elevated stroke incidence rates, potentially two to three times higher than those observed in Western Europe and the United States [3]. The primary impediments to healthcare accessibility in Africa include financial constraints, geographical distances, and prolonged waiting times, with specific challenges highlighted in Zambia's remote districts [5, 6]. Telestroke plays a crucial role in stroke care systems, facilitating rapid assessment of individuals with acute stroke symptoms in remote or underserved regions. It serves as a pivotal tool for identifying optimal candidates for hospital transfer, effectively minimizing unnecessary transfers of patients, especially those with mild stroke syndromes or stroke mimics, who can be appropriately managed at local facilities [7]. The paper assesses the advantageous impact of telestroke services on stroke management, encompassing clinical outcomes, patient satisfaction, and healthcare accessibility, with the goal of drawing lessons for Africa.

The utilization of telestroke in stroke management is deemed safe and showcases enhancements in functional outcomes [9]. A comprehensive systematic review, encompassing 19 studies with a collective participation of 28,496 subjects and evaluating both prehospital and in-hospital telestroke interventions, revealed a substantial increase in the proportion of patients treated within a 3-h window (OR 2.15; 95% CI 1.37–3.40; I2 = 0%) and improved 3-month functional outcomes (OR 1.29; 95% CI 1.01–1.63; I2 = 44%) without a simultaneous rise in the rate of symptomatic intracranial hemorrhage (OR 1.27; 0.65–2.49; I2 = 0%). Also, telestroke interventions were associated with a reduction in onset-to-treatment time (mean difference: −27.97 min; 95% CI −35.51, −20.42; I2 = 63%) and a decreased in-hospital mortality rate (OR 0.67; 95% CI 0.52–0.87; I2 = 0%) [13]. The application of telestroke has the potential to streamline consultations by stroke neurologists, determining the suitability of administering thrombolysis therapy. This approach could enhance utilization rates and the efficiency of the thrombolysis administration timeline in managing strokes, especially in diabetic patients [10]. In an observational study conducted in western China, involving 11,449 admissions predominantly diagnosed with ischemic stroke, the percentage of patients undergoing intravenous thrombolysis increased from 6.7% to 7.4% after the integration of telestroke. Furthermore, the mean door-to-needle time (DNT) significantly decreased following the implementation of the telestroke network (63.76 ± 13.50 vs. 52.66 ± 25.49 min; *p* < 0.001) [14].

Telestroke plays a crucial role in addressing time-sensitive decisions regarding intravenous thrombolysis (IVT) for acute ischemic stroke patients, especially in hospitals lacking on-site neurologists. A study conducted in Germany reported a notable 14.9% IVT rate within telestroke networks, underscoring their positive impact [15]. A prospective observational study assessing the East of England Stroke Telemedicine Partnership's utilization and effectiveness in providing hyperacute stroke care demonstrated an increase in thrombolysis rates from 38.8% in 2014 to 45.9% in 2019 posttelestroke implementation. Additionally, the median (interquartile range) time for thrombolysis decreased from 10 h (prethrombolysis) to 6 h [16]. Telestroke has exhibited high diagnostic accuracy in distinguishing between stroke and its mimics. A retrospective analysis covering all evaluations within Ochsner Health's Telestroke program from April 2015 to April 2016 in the United States indicated accurate diagnoses in 85% of the 874 evaluations. The sensitivity, specificity, positive predictive value, and negative predictive value were determined to be 97.8%, 82.5%, 93.7%, and 93.4%, respectively. These findings strongly support the claim of elevated diagnostic accuracy in telestroke consultations [12].

Telestroke services play a pivotal role in increasing the likelihood of prompt and consistent healthcare access for patients residing in medically underserved regions. This effectively addresses challenges arising from a scarcity of physicians, a factor often contributing to delayed diagnoses and treatment. Furthermore, telestroke contributes to improving continuity of care by minimizing unnecessary patient transfers and redundant testing [17]. In India, where only 2.67% of neurologists and neurosurgeons are in rural areas, serving a population of 84.59 million, the introduction of telemedicine, including telestroke, has proven instrumental in alleviating the critical shortage of healthcare professionals. The effective utilization of commercially deployed video conferencing (VC) systems facilitates teleconsultation sessions for stroke patients, offering a practical solution to enhance healthcare accessibility [18].

The implementation of telestroke services elevates patient satisfaction. In an 18-month study, assessing 186 telestroke users, 76% expressed high satisfaction, with significant correlations found in technology ratings (*p* < 0.0001), favorable telepresence evaluations (*p* < 0.0001), and positive provider communication assessments (*p* < 0.0001). Among the fully satisfied patients, an impressive 97% were willing to recommend telestroke to others experiencing stroke symptoms. Interestingly, even among the 44 patients with some dissatisfaction, 69% expressed an inclination to recommend the service, emphasizing the perceived value of telestroke [19]. In another study that analyzed 69 patients engaged in neurology follow-up visits via telestroke, the Telestroke Patient Satisfaction Measure indicated a mean score of 55, with an average of 94% of responses showing agreement. The Consultation and Relational Empathy score averaged 44, with an impressive 90% of responses rated as “*very good*” or “*excellent*.” Participating neurologists acknowledged the significant enhancement of work-life balance through reduced travel time, effectively bridging the gap between patients' local care needs and physicians' need for improved equilibrium [20].

The introduction of telestroke CT services plays a pivotal role in ensuring equal access to high-quality stroke healthcare. A qualitative study conducted in Hallingdal, Norway, revealed that participants experienced a sense of safety and reassurance with the telestroke CT service. They felt secure, knowing that medical assistance was readily available in proximity in case of a stroke for themselves or their loved ones [21]. The integration of telestroke in acute care treatments and stroke prevention has led to a decline in mortality among stroke survivors, causing a shift in the United States ranking from the third to the fifth leading cause of death [22].

Healthcare systems in Africa may learn lessons from the successful implementation of telestroke, with a focus on both external and internal factors that have proven instrumental in the adoption of telestroke in countries like the United States. Key external factors include market competition and critical access hospital status, while internal factors encompass payer mix, patient volume, and hospital profitability [23]. Drawing insights from the United States, African healthcare systems may benefit from understanding the efforts of US healthcare authorities in advancing regulatory science for digital health. Notably, the 2017 Digital Health Innovation Action Plan and the subsequent establishment of the Digital Health Center of Excellence (DHCoE) in 2020 underscore the commitment to this cause [24].

The successful deployment of telestroke services in Africa is impeded by a myriad of ethical, technological, regulatory, and infrastructural challenges. Ethical concerns surrounding doctor–patient privacy, confidentiality, informed consent, and data security remain significant barriers, as they contribute to the potential failure of digital health interventions, including telestroke [31]. The virtualization of patient care is often perceived as dehumanizing, which may deter its adoption within many health systems in Africa. Technological prerequisites such as comprehensive network coverage, stable internet connectivity, and affordable data services are critical for effective telestroke implementation. However, many African countries, particularly in rural areas, face persistent limitations in network infrastructure, unreliable internet access, and high data costs, severely constraining the widespread adoption of telestroke solutions [26]. Additionally, the absence of a robust governance framework to harmonize the interests of key stakeholders such as governmental bodies, private sector entities, nongovernmental organizations, and local communities has resulted in sustainability challenges, particularly following the conclusion of externally funded project cycles. The heavy reliance on external funding highlights a critical deficiency in strategic planning for the long-term viability of telestroke solutions [27]. Regulatory inconsistencies across African countries further exacerbate these challenges, creating ambiguity and impeding the standardization of telestroke services. The lack of clear guidelines discourages healthcare providers from integrating telestroke into routine clinical practice [30]. Also, a shortage of healthcare professionals with the necessary hybrid expertise to operate telestroke systems further hinders effective implementation, with insufficient clinician buy-in observed in countries such as Ghana, Nigeria, and Mali [29]. CT scanners and MRI machines are vital for the early detection and accurate diagnosis of strokes within telestroke systems, but their high costs often limit their accessibility and widespread use in many African healthcare facilities [25].

To address the identified limitations and facilitate the effective implementation of telestroke services in Africa, a multifaceted and strategic approach may be considered. Policymakers may prioritize the development of standardized regulatory frameworks that provide clear guidelines on ethical, legal, and operational aspects of telestroke, ensuring compliance with data security, patient confidentiality, and informed consent requirements. Valuable lessons can be drawn from Zambia's “Guidelines for the Quality Assurance of Telemedicine Services,” which provide comprehensive instructions for provider-to-provider and client-to-provider telestroke interactions while ensuring adherence to established scopes of practice. These guidelines mandate that telestroke providers hold valid licenses and accreditation certificates, thereby ensuring that only qualified professionals deliver these critical services [28, 29]. Strengthening network infrastructure, expanding broadband coverage, and implementing cost-reduction strategies for data services, particularly in rural areas, may enhance technological accessibility and improve the reliability of telestroke solutions. Sustainable financing mechanisms, including the integration of telestroke into national healthcare budgets and the promotion of public-private collaborations, may reduce dependency on external funding and ensure long-term viability. Capacity-building initiatives focusing on equipping healthcare professionals with both clinical and digital competencies may enhance the adoption of telestroke technologies, particularly in countries such as Ghana, Nigeria, and Mali, where clinician buy-in remains limited. Drawing on Zambia's telemedicine guidelines, other African nations may establish comprehensive policies to regulate provider-to-provider and client-to-provider interactions while ensuring that only accredited professionals deliver telestroke services. Additionally, targeted community engagement and awareness campaigns may be implemented to address misconceptions surrounding the dehumanization of virtual care, fostering greater acceptance and trust among healthcare providers and patients. A coordinated effort among governments, healthcare institutions, private sector stakeholders, and international organizations may be essential in creating a resilient and scalable telestroke ecosystem across the continent. Devices such as the Strokefinder MD100, Transcranial Doppler ultrasound, and Lucid represent portable CT and MRI solutions that are relatively less expensive, enabling the detection of hemorrhages, measurement of cerebral blood flow velocity, and identification of patients with large vessel occlusions (LVOs), respectively [25]. These imaging devices may be integrated with telestroke platforms in Africa to facilitate the instantaneous sharing and analysis of essential medical data, thereby ensuring that patients receive appropriate treatment without undue delays.

## 5. Conclusion

In conclusion, this paper underscores the significant impact of telestroke services on stroke management, covering clinical outcomes, patient satisfaction, and healthcare accessibility. However, it is essential to acknowledge the limitations of this study, which may affect the generalizability of the findings. The reliance on a limited number of studies and the strict exclusion and inclusion criteria, limiting the analysis to peer-reviewed journals and English-language publications, may restrict the broader applicability of the results. Despite these constraints, the research highlights the transformative potential of telestroke in enhancing stroke care systems. It is recommended that future studies incorporate a more diverse range of sources and languages to provide a comprehensive understanding of telestroke's global implications. Considering the unique healthcare landscape of Africa, it is suggested that healthcare systems in the region learn from the successful implementation of telestroke in other countries. External and internal factors, such as market competition, critical access hospital status, payer mix, patient volume, and hospital profitability, should be carefully examined to tailor telestroke integration to Africa's specific needs. The experiences of US healthcare authorities in advancing regulatory science for digital health, as exemplified by the Digital Health Innovation Action Plan and the DHCoE, offer valuable insights for African nations to develop their regulatory frameworks. Collaboration with international organizations and investment in infrastructure, coupled with educational initiatives, can further accelerate the adoption and successful implementation of telestroke in Africa.

## Figures and Tables

**Table 1:** Databases, number of identified and included studies, and percentages of included studies.

**Electronic databases**	**Number of identified studies**	**Number of included studies**	**% of included studies**
Scopus	121	6	19.4
PubMed	172	9	29.0
ProQuest	293	12	38.7
Google Scholar	295	4	12.9

**Table 2:** Summary characteristics of included studies.

**Author**	**Year**	**Country**	**Topic**	**Study design**	**Key findings**
Roth et al. [1]	2020	United States	Global Burden of Cardiovascular Diseases and Risk Factors, 1990–2019	Epidemiological study	Cerebrovascular accidents, commonly known as strokes, are a leading contributor to worldwide mortality and a substantial contributor of incapacitation. The global burden of stroke in 2019 was 143 million recorded cases

Ansari et al. [2]	2023	Iran	Telestroke: A Novel Approach for Post-Stroke Rehabilitation	An overview	Preponderantly, low- and middle-income countries bear the most substantial global burden of strokes. Swift identification and treatment of afflicted individuals, particularly in remote or rural locales, are imperative measures to mitigate ensuing complications

Akinyemi et al. [3]	2021	Nigeria	Stroke in Africa: profile, progress, prospects and priorities	Scoping review	Approximately 87% of disability arising from strokes and 70% of stroke-related fatalities occur in low-income and middle-income nations. On a global scale, an estimated one in four adults is projected to experience a stroke during their lifetime. Africa may exhibit stroke incidence rates two to three times higher than those observed in Western Europe and the United States, coupled with elevated stroke prevalence

Owolabi et al. [4]	2018	Nigeria	The epidemiology of stroke in Africa: A systematic review of existing methods and new approaches	Systematic review	Africa shoulders the predominant burden of hypertension, identified as the most robust and prevalent modifiable risk factor for strokes. In the African context, stroke emerges as a pivotal health determinant, historically deemed infrequent due to resource constraints hindering comprehensive community-based studies for accurate burden assessment

Kyei-Nimakoh et al. [5]	2017	Australia	Access barriers to obstetric care at health facilities in sub-Saharan Africa—a systematic review	Systematic review	Primary impediments to healthcare service accessibility in Africa encompass the financial outlay associated with services, the geographical distance between health facilities and the residences of service users, and prolonged waiting time at healthcare facilities

Mweemba et al. [6]	2021	Zambia	Access barriers to maternal healthcare services in selected hard-to-reach areas of Zambia: a mixed methods design	Mixed methods design	In a concurrent mixed methods investigation carried out in Zambia's challenging-to-reach districts of Kaputa and Ngabwe, findings revealed that residents had to traverse approximately 12 kilometers to reach healthcare services. The study indicated that patients were inclined to remain at home rather than seek health services when lacking transportation fares. Beyond the distance to health centers, participants raised concerns about the poor condition of roads, exacerbated during rainy seasons, rendering modes of transport such as motorcycles and cars challenging

Harahsheh et al. [7]	2022	United States	Telestroke's Role Through the COVID-19 Pandemic and Beyond	Scoping review	Telestroke is a pivotal element within stroke systems of care, offering a means to swiftly assess individuals exhibiting acute stroke symptoms in remote or underserved regions. Telestroke serves as a crucial tool in identifying optimal candidates for hospital transfer. Its application effectively limits unwarranted transfers of patients, particularly those with mild stroke syndromes or stroke mimics, who can be appropriately managed at local facilities

Jun-O'Connell et al. [8]	2023	United States	Outcomes of Telestroke Inter-Hospital Transfers Among Intervention and Non-Intervention Patients	Exploratory study	Telestroke is the use of remote information and communication technologies to enable the delivery of stroke care for patients, facilitating the remote practice of physicians as stroke patients concurrently receive care from a distance (Connell et al., [8])

Mohamed et al. [9]	2023	Canada	Is telestroke more effective than conventional treatment for acute ischemic stroke? A systematic review and meta-analysis of patient outcomes and thrombolysis rates	Systematic review	The use of telestroke for managing strokes is considered safe and demonstrates improvement in functional outcomes

Sharrief et al. [11]	2023	United States	Telehealth Trials to Address Health Equity in Stroke Survivors	A review	The integration of telestroke services into transitional and post-stroke care has significant potential to bridge critical gaps in the continuum of care for stroke survivors, who often encounter challenges distinct from those faced by individuals with other chronic conditions. Telestroke alleviates transportation challenges, particularly for patients subject to driving restrictions, thereby enhancing accessibility and continuity of care in this vulnerable population. By addressing mobility limitations and reducing dependence on specialized equipment required to access physical clinic spaces, telestroke minimizes logistical barriers to care

Lazarus et al. [13]	2020	Indonesia	Telestroke strategies to enhance acute stroke management in rural settings: A systematic review and meta-analysis	Systematic review	A systematic review that assessed 19 studies with a collective participation of 28,496 subjects, inclusive of prehospital and in-hospital telestroke interventions, revealed a notable increase in the proportion of patients treated within a 3-h window (OR 2.15; 95% CI 1.37–3.40; I2 = 0%) and improved three-month functional outcomes (OR 1.29; 95% CI 1.01–1.63; I2 = 44%) without a concurrent rise in the rate of symptomatic intracranial hemorrhage (OR 1.27; 0.65–2.49; I2 = 0%). Moreover, telestroke interventions were associated with a reduction in onset-to-treatment time (mean difference −27.97 min; 95% CI −35.51, −20.42; I2 = 63%) and a decreased in-hospital mortality rate (OR 0.67; 95% CI 0.52–0.87; I2 = 0%)

Nathaniel et al. [10]	2019	United States	The telestroke and thrombolysis therapy in diabetic stroke patients	Retrospective study	Telestroke could potentially facilitate prompt consultations by stroke neurologists to determine the appropriateness of administering thrombolysis therapy. Such an approach may enhance the utilization rates and efficiency of the thrombolysis administration timeline in the management of stroke in diabetic patients

Chen et al. [14]	2022	China	Telestroke for the Treatment of Ischemic Stroke in Western China During the COVID-19 Pandemic: A Multicenter Observational Study	Multicenter observational study	In an observational study conducted in western China, encompassing 11,449 admissions primarily diagnosed with ischemic stroke, the percentage of patients undergoing intravenous thrombolysis rose from 6.7% to 7.4% following the integration of telestroke. Moreover, the mean door-to-needle time (DNT) exhibited a significant reduction after the implementation of the telestroke network (63.76 ± 13.50 vs. 52.66 ± 25.49 min; *p* < 0.001)

Worthmann et al. [15]	2023	Germany	Telestroke networks for area-wide access to endovascular stroke treatment.	Narrative review	Telestroke networks address the critical need for time-sensitive decisions regarding intravenous thrombolysis (IVT) in acute ischemic stroke patients, particularly in hospitals without on-site neurologists. A study in Germany reported a 14.9% IVT rate within telestroke networks, highlighting the positive impact of such networks

Evans et al. [16]	2022	United Kingdom	Hyperacute stroke thrombolysis via telemedicine: a multicentre study of performance, safety and clinical efficacy	Multicentre study	In a prospective observational study examining the utilization and effectiveness of the East of England Stroke Telemedicine Partnership in delivering hyperacute stroke care, findings revealed an increase in thrombolysis rates from 38.8% in 2014 to 45.9% in 2019 following the implementation of telestroke. The median (interquartile range) time for thrombolysis decreased from 10 h (prethrombolysis) to 6 h

Poongkunran et al. [12]	2023	United States	Diagnostic accuracy of telestroke consultation: a Louisiana-based tele-network experience	Retrospective study	Telestroke has demonstrated a high diagnostic accuracy in discerning between stroke and its mimics. In a retrospective analysis encompassing all evaluations within Ochsner Health's Telestroke program from April 2015 to April 2016 in the United States, accurate diagnoses were achieved through telestroke consultations in 85% of the 874 evaluations conducted. The sensitivity, specificity, positive predictive value, and negative predictive value were determined to be 97.8%, 82.5%, 93.7%, and 93.4%, respectively. These findings affirm the assertion of elevated diagnostic accuracy in telestroke consultations

Ali et al. [17]	2020	United States	Role of Artificial Intelligence in Telestroke: An Overview.	An overview	Telestroke services offer patients residing in medically underserved regions a higher likelihood of accessing prompt and consistent healthcare. This mitigates challenges associated with a relative scarcity of physicians, which frequently results in delayed diagnoses and treatment. Additionally, telestroke aids in enhancing continuity of care by minimizing unnecessary patient transfers and redundant testing

Kissi et al. [18]	2023	Ghana	Telehealth services for global emergencies: implications for COVID-19: a scoping review based on current evidence	Scoping review	In India, despite only 2.67% of neurologists and neurosurgeons residing in rural areas, serving a population of 84.59 million, the introduction of telemedicine, including telestroke, has been instrumental in mitigating the critical shortage of healthcare professionals. Commercially deployed video conferencing (VC) systems are effectively utilized to facilitate teleconsultation sessions for patients with stroke conditions (Kissi et al., [18]).

Lyerly et al. [19]	2021	United Kingdom	Provider Communication and Telepresence Enhance Veteran Satisfaction with Telestroke Consultations.	Qualitative study	In a study that assessed the satisfaction of 186 telestroke users over an 18-month period, 142 (76%) participants expressed high satisfaction with telestroke. Factors significantly correlated with patient contentment included elevated technology ratings (*p* < 0.0001), favorable telepresence evaluations (*p* < 0.0001), and positive provider communication assessments (*p* < 0.0001). Of the 142 patients reporting full satisfaction with the telestroke service, an impressive 97% expressed their willingness to recommend telestroke to other patients experiencing stroke symptoms. Intriguingly, among the 44 patients expressing less than full satisfaction with the consultation, 69% still indicated their inclination to recommend the service

Truitt et al. [20]	2023	United States	Patient Satisfaction and Perception of Physician Empathy in Outpatient Community General Neurology Telemedicine	Prospective cohort study	69 patients engaged in neurology follow-up visits via telestroke, the Telestroke Patient Satisfaction Measure indicated a mean score of 55, with an average of 94% of responses showing agreement. The Consultation and Relational Empathy score averaged 44, with an impressive 90% of responses rated as “*very good*” or “*excellent*.” Participating neurologists acknowledged the significant enhancement of work-life balance through reduced travel time, effectively bridging the gap between patients' local care needs and physicians' need for improved equilibrium

Kjelle and Myklebust [21]	2022	Norway	Implementation of a telemedicine, stroke evaluation service; a qualitative study	Qualitative study	The implementation of telestroke CT services ensures equitable access to high-quality stroke healthcare. According to findings from a qualitative study conducted in Hallingdal, Norway, participants expressed a sense of safety and reassurance with the telestroke CT service. They felt secure knowing that medical assistance was readily available in close proximity in case they or their loved ones experienced a stroke

Naqvi et al. [22]	2022	United States	TASC (Telehealth After Stroke Care): a study protocol for a randomized controlled feasibility trial of telehealth-enabled multidisciplinary stroke care in an underserved urban setting	Parallel two-armed feasibility randomized controlled trial	Improvement in acute care treatments and prevention of strokes using telestroke have contributed to decreased mortality among stroke survivors, taking stroke from the third to the fifth leading cause of death in the United States

Shea et al. [23]	2018	United States	Telestroke Adoption among Community Hospitals in North Carolina: a Cross-sectional Study	Cross-sectional study	The adoption of telestroke services is significantly correlated with both external factors, such as market competition and critical access hospital status, and internal hospital factors, including payer mix, patient volume, and hospital profitability

Busti et al. [24]	2021	Italy	Telestroke: Barriers to the Transition	Narrative review	Africa may draw insights from the efforts of US healthcare authorities in advancing regulatory science for digital health. This commitment is elucidated in the 2017 Digital Health Innovation Action Plan and was further reinforced in 2020 with the establishment of the Digital Health Center of Excellence (DHCoE)

Patil et al. [25]	2022	Ireland	Detection, Diagnosis and Treatment of Acute Ischemic Stroke: Current and Future Perspectives	Narrative review	CT scanners and MRI machines are vital for the early detection and accurate diagnosis of strokes within telestroke systems, but their high costs often limit their accessibility and widespread use in many African healthcare facilities. Devices such as the Strokefinder MD100, transcranial Doppler ultrasound, and Lucid represent portable CT and MRI solutions that are relatively less expensive, enabling the detection of hemorrhages, measurement of cerebral blood flow velocity, and identification of patients with large vessel occlusions (LVOs), respectively. These imaging devices must be integrated with telestroke platforms to facilitate the instantaneous sharing and analysis of essential medical data, thereby ensuring that patients receive appropriate treatment without undue delays

Chitungo et al. [26]	2021	Zimbabwe	Utility of telemedicine in sub-Saharan Africa during theCOVID-19 pandemic. A rapid review	Rapid review	Successful telestroke implementation requires comprehensive network coverage, reliable internet connectivity, and affordable data bundles. In Africa, these technological prerequisites are often hindered by limited network infrastructure, inconsistent internet access, and the high cost of data services, particularly in rural areas. These challenges significantly impede the adoption and effective utilization of telestroke solutions across the continent

Dodoo et al. [27]	2022	Ghana	The development of telemedicine programs in Sub-Saharan Africa: Progress and associated challenges	Systematic review	The implementation of telestroke in Africa has been significantly hindered by the absence of a robust framework to harmonize the interests of diverse stakeholders. Existing telestroke initiatives have encountered persistent sustainability challenges, particularly following the conclusion of project funding cycles. Moreover, the heavy reliance on external financial support has highlighted a critical deficiency in strategic planning for long-term viability, with minimal integration of key stakeholders, including governmental bodies, private sector entities, non-governmental organizations, and local communities, to ensure sustained impact and operational continuity

Muyunda and Mpundu [28]	2023	Zambia	Mapping the Regulatory Framework for Telemedicine in Zambia: A Content Analysis	Content analysis	Lessons can be drawn from Zambia's “Guidelines for the Quality Assurance of Telemedicine Services,” issued by the Health Professions Council. These guidelines offer comprehensive instructions for both provider-to-provider and client-to-provider telestroke interactions while maintaining the established scopes of practice for all health professionals. Additionally, the guidelines require telestroke providers to hold valid licenses and accreditation certificates, ensuring that only qualified and authorized individuals deliver these essential services

Dodoo et al. [29]	2021	Ghana	Telemedicine use in Sub-Saharan Africa: Barriers and policy recommendations for COVID-19 and beyond	Systematic review	Skilled healthcare professionals capable of operating telestroke systems are essential for their effective implementation. However, most medical facilities in Africa lack professionals with the necessary hybrid (physical and electronic) expertise to utilize telestroke medical equipment. For instance, there is insufficient initial telestroke buy-in from clinicians in countries such as Ghana, Nigeria, and Mali, further hindering the adoption and success of telestroke initiatives across the continent

Ayo-Farai et al. [30]	2023	Nigeria	Telemedicine in Health Care: A Review of Progress and Challenges in Africa	A review	Diverse regulatory frameworks across African countries create significant ambiguity, impeding the establishment of uniform telestroke standards. The absence of clear, cohesive guidelines discourages healthcare providers from adopting and integrating telestroke services into routine practice

Mbunge et al. [31]	2022	Eswatini	Are we there yet? Unbundling the potential adoption and integration of telemedicine to improve virtual healthcare services in African health systems	Systematic review	The deployment of telestroke services raises significant ethical concerns, particularly with respect to doctor-patient privacy, confidentiality, informed consent, and data security, which are critical factors contributing to the potential failure of digital health interventions like telestroke. Also, the virtualization of patient care is often perceived as a form of dehumanization, which may hinder the adoption and integration of telestroke services within many health systems

## Data Availability

Pieces of the literature analysed during the current study are available online and can also be made available through the corresponding author upon request.
